# Wood-PHA Composites: Mapping Opportunities

**DOI:** 10.3390/polym10070751

**Published:** 2018-07-07

**Authors:** Luigi-Jules Vandi, Clement Matthew Chan, Alan Werker, Des Richardson, Bronwyn Laycock, Steven Pratt

**Affiliations:** 1 School of Chemical Engineering, University of Queensland, St. Lucia, QSD 4072, Australia; c.chan@uq.edu.au (C.M.C.); alan.werker@promiko.se (A.W.); b.laycock@uq.edu.au (B.L.); s.pratt@uq.edu.au (S.P.); 2 Promiko AB, 23442 Lomma, Sweden; 3 Norske Skog Paper Mills (Australia) Ltd., Boyer, TAS 7140, Australia; des.richardson@norskeskog.com

**Keywords:** polyhydroxyalkanoates, PHA, PHBV, biocomposites, wood plastic composites, WPC, economics, applications, market study

## Abstract

Polyhydroxyalkanoate (PHA) biopolymers are emerging as attractive new sustainable polymers due to their true biodegradability and highly tuneable mechanical properties. However, despite significant investments, commercialisation barriers are hindering the capacity growth of PHA. In this work, we investigated the market potential for wood plastic composites (WPCs) based on PHAs. We considered the latest global production capacity of PHAs, estimated at 66,000 tonnes/year, and examined the implications of using PHAs for WPC production on the WPC market. Results indicate that a hypothetical usage of the current global PHA production for WPC manufacture would only represent the equivalent of 4.4% of the global WPC market, which is currently experiencing a 10.5% compounded annual growth rate. An economic assessment revealed that a wood-PHA composite as a drop-in alternative WPC product could cost as little as 37% of the cost of its neat PHA counterpart. Thus, WPCs with PHA offer a means to access benefits of PHA in engineering applications at reduced costs; however, further developments are required to improve strain at failure. The successful adoption of wood-PHA composites into the market is furthermore reliant on support from public sector to encourage biodegradable products where recycling is not a ready solution.

## 1. Introduction

The scale of global polymer production, which ramped up to 322 million tonnes/year in 2015 [1], is raising serious concerns regarding end-of-life disposal and plastic contamination of the environment. Between 22% and 43% of polymers used worldwide are disposed of in landfill, and approximately 10–20 million tonnes of plastic ends up in oceans each year [2]. Governments are gradually legislating for a stronger emphasis on the circular economy, by introducing bans on landfills and enforcing the use of bioplastics for certain applications, such as shopping bags. Bioplastics are, by definition, sourced from a renewable resource (biosourced) and/or biodegradable. Amongst the bioplastics, polyhydroxyalkanoates (PHAs) have attracted significant interest as promising new sustainable biopolymers due to their combined low environmental impact (true biodegradability and non-toxicity) and high functionality (tuneable mechanical and physical properties). 

Commercialisation of PHAs is still in its early stages, and although its global production capacity is one of the fastest growing amongst biopolymers [3], small capacities and volumes are associated with relatively higher costs, which makes it challenging to compete up-front on just price with petroleum-based plastics that are produced on a very large scale. As a relatively new family of polymers, PHAs need to gain further end-user acceptance and grow their own market, including for target/niche applications. 

Potential applications for PHAs include, consumer goods, agriculture, packaging, furniture, and personal care products. Within those applications, the use of PHA to make Wood Plastic Composites (WPCs) represents one option, which is explored in the present work. WPCs have gained a substantial market in the last decade, due to transformative technological advancements in compounding and processing. The WPC global market was as high as 3.0 million tonnes/year in 2014, and is forecast to double by 2020 [4]. The use of PHA in combination with wood reinforcement to manufacture WPCs will help solve a growing issue with end-of-life disposal of traditional WPCs, by replacing the non-degradable petroleum-based matrix with fully biodegradable PHA. 

However, questions remain in the markets for these new materials as well as their cost-effectiveness and the feasibility for production on a large scale. In this paper, we present an up-to-date overview of commercialisation activities and the emerging trends as inferred from intellectual property developments generated around PHAs. We outline observed properties of wood-PHA composites coupled to the material potential. We discuss application opportunities and markets for these novel biobased WPC-materials, and look at the current PHA production capacity in detail. Finally, we present an illustrative economic assessment of wood-PHA composites, and look at potential cost savings beyond their lower raw material costs. 

## 2. PHA and Wood-PHA Composites

### 2.1. PHA and Its Commercialisation

Polyhydroxyalkanoates (PHAs) form a family of naturally occurring polyesters, which are produced microbiologically. Material properties range from rigid thermoplastics to stretchy elastomers, and can be controlled by the choice of substrate, bacteria and fermentation conditions [5]. Some of the more common PHAs include polyhydroxypropionate (PHP), poly(3-hydroxybutyrate) (PHB), poly(3-hydroxyvalerate) (PHV), poly(3-hydroxyhexanoate) (PHH), poly(3-hydoxyoctanoate) (PHO), etc., and co-polymers thereof [6,7,8]. The main variant of these bio-polyesters is the homopolymer PHB, which is relatively highly crystalline and has similar properties to conventional polyesters such as polyethylene (PE) and polypropylene (PP). However, PHB demonstrates low nucleation density and consequently tends to form a microstructure with large spherulites, which are known to impair the mechanical properties, most notably toughness. To overcome these drawbacks, various copolymers and co-polymer blends, including poly(hydroxybutyrate-*co*-hydroxyvalerate) (PHBV), poly(hydroxybutyrate-*co*-hydroxyhexanoate) (PHBHx), and poly(hydroxybutyrate-*co*-hydroxyoctanoate) (PHBO), have been investigated [9]. 

PHAs offer unique benefits for end of life disposal, in line with the increasing government restrictions on landfills and the strong drive to a circular economy in Europe. PHAs are both compostable and biodegradable in soil, riverine and marine environments. Other biodegradable polymers such as polylactic acid (PLA) are compostable, but remain in marine environments for up to a thousand years [10]. This distinction in properties and conditions for biodegradation is important to note, considering polymer pollution in the marine environment is particularly harmful and is estimated at 100 million tonnes of polymer in total, causing a cost burden of ecosystem service damage of approximately 13 billion USD per year [11]. Notwithstanding, polymers generally compete commercially on the market based on the up-front price without consideration of the downstream burden. 

The combined advantages of flexible mechanical properties with a lower end-of-life environmental impact are unique to PHAs, which makes them well suited to genuinely meet the requirements of a circular bioeconomy. 

**Figure 1:**
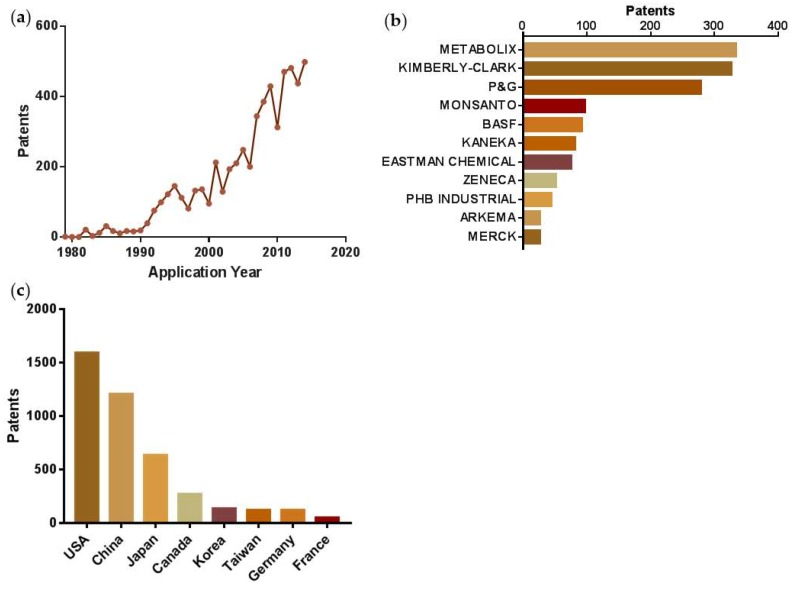
Patent applications related to poly(hydroxybutyrate-*co*-hydroxyvalerate) ‘PHBV’, showing. (**a**) application trends over time; (**b**) major assignees; and (**c**) major authorities.

This triggered the initial commercialisation efforts of the PHBV copolymer in the late 1980s, with early patents being filed by Metabolix Inc. (Woburn, MA, USA) in the 1990s. Subsequently, the first generation of commercial PHA bioplastics, with unrivalled biodegradation properties, was developed [10]. This no doubt stimulated the emerging interest in bioplastics production, resulting in a number of large companies entering the market. Figure 1 shows patent application trends (Figure 1a) and the major assignees (Figure 1b) related to PHBV biopolymer (the most common type of PHA) intellectual property developments over the past 40 years. These trends show a progressive increase in patent applications, which have been dominated by large multinational companies and a few small and medium-sized enterprises (SMEs), including Metabolix, Kimberly-Clark, The Procter & Gamble Company (P&G), Monsanto, BASF, Kaneka, Eastman Chemical, Zeneca, PHB Industrial, Arkema and MERCK. Together, the top 10 companies span the markets of consumer goods, polymers, personal care, agriculture, chemicals, and pharmaceuticals. The development of a wide spectrum of applications is predominantly found in the USA, China and Japan, followed by Canada, Korea, Taiwan, Germany, and France (Figure 1c). However, despite the significant range of intellectual property (IP) filings with underlying research and development efforts, production scales, commercialisation, and the market of PHA products remains at an early stage. 

Global production is hindered and this limits the extent and opportunity for the development of PHA-based products. In contrast to a 10% annual growth between 2012 and 2014, the bio-based polymer production capacity growth data now shows a 4% annual growth rate from 2014 to 2016, which is almost the same as for the overall global polymer production [3]. Although PHA exhibits the fastest growth rate, the overall lower than expected annual growth rate of bio-based polymers has been reported to be due to several factors [3,5]: (1) the low oil prices, (2) an unfavourable political framework in most countries, (3) a slower than expected growth rate of the capacity utilisation, and (4) societal concern in the adoption of new bio-products. Examples for the latter include genetically modified feedstock, biodiversity loss through monocultures, rainforest depletion by palm oil imports, and the debate about the use of food crops to feed chemicals for industrial applications. These are all examples of the importance of trust in bioproducts by end-users and risks when a raw material production is judged within restricted assumed context. However, better communication with end-users, through certifications and quality labels, is expected to assist growth in successful market introduction. In the case of PHA, we postulate that the main two commercialisation barriers are: (1) the production cost of PHA compared to petroleum-based polymers (and indirectly to oil prices), and (2) hindered market adoption due to limited consumer experience with these materials. Both of these factors work against investment in production, which would, in turn, establish supply chains that promote the discovery of benefits and opportunity in market experience. Markets that do not exist cannot be analysed and predicted with any kind of confidence. In the end, it has been repeatedly shown that suppliers and customers must ultimately discover them together [12]. In the present work, we consider the use of novel PHA-based WPCs, as one potential market entry point for PHA-based materials. 

### 2.2. Wood-PHA Composites as Novel WPCs

**Figure 2:**
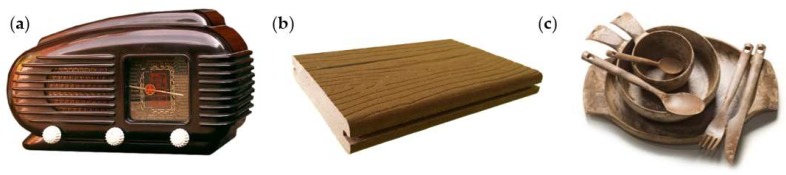
Example of Wood Plastic Composites (WPC) products. (**a**) a 1953 Tesla Talisman radio from Bakelite; (**b**) Karle and Rubner decking board from white oak wood fibre and polyethylene (PE); (**c**) Kupilka plate and cutlery set from 50% pine wood fibre and 50% polypropylene (PP).

Wood plastic composites (WPCs) refer to any composites that contain wood (of any form) and a polymer (thermoset or thermoplastic). The first WPCs date back to the early 1900s, and were composed of phenol-formaldehyde (a thermoset) and wood flour, marketed under the trade name Bakelite. Today, most WPCs focus on wood-thermoplastic composites, with the general understanding that the ‘plastic’ is a thermoplastic. Examples of WPC products are shown in Figure 2. The growth of the WPC industry involved the interfacing of two industries that historically knew little about each other, and had very different expertise and perspective. The plastics industry has knowledge of plastics processing, and the forestry products industry has more experience and resources in the building and paper product markets. Thus, plastic processors often lacked knowledge about wood, and were reluctant to replace existing fillers (glass, or carbon) with wood fibres, even though they are from a renewable resource, and are less expensive, lighter and less abrasive to processing equipment. The main concerns associated with wood flour/fibres were low bulk density (difficulty to feed into typical plastic processing equipment), low thermal stability above 200 °C, and tendency to absorb moisture [13]. However, these issues were gradually resolved [14]. Thermoplastics were selected that melt or could be processed at temperatures below the thermal degradation temperatures of the wood fibres, such as (in order of market share) specific grades of polypropylene (PP), polyethylene (PE), and polyvinyl chloride (PVC) [15]. Technological advancements were made in compounding, which facilitated processing and minimised moisture uptake. For instance, a novel patented technology by Scion (Rotorua, New Zealand) can convert high temperature thermomechanical pulp (TMP) fibres into a feedstock that can be readily used by the plastic industry, which was commercialised under the tradename Woodforce [16]. Another proprietary technology developed by Jeluplast (Rosenberg, Germany) consists of mixing wood fibres and the thermoplastic at elevated temperature and pressure to produce a uniform granulate in which each individual wood fibre is fully encapsulated in the polymer [17]. This method enables superior homogeneity and ease of processing and allows for wood contents of up to 70% to be achieved [18]. The WPC market has, since then, grown dramatically to a total volume of 3 million tonnes/year in 2014, and with expectation to double by 2020 [4].

With the WPC market growth [4], end-of-life disposal/recovery concerns also increase. Fully recyclable products previously made entirely from PP or PE are now being replaced with WPCs, which are non-recyclable through conventional methods. Still, WPCs are marketed as recyclable products. However, a specific waste legislation classification for WPCs does not exist. The recycling depots face increasing quantities of WPCs that cannot be allocated to a particular area [19]. This issue is primarily due the combination of a renewable and biodegradable material (wood fibres) with a petroleum-based and non-degradable material (e.g., PP, PE), which traditionally require different routes for end-of-life management. In practice, only ‘internal recycling’, in which production residue is re-used during the manufacturing process, is being carried out by WPC manufacturers. According to one study [19], very limited investigations have been carried out for WPCs on their ‘end-of-life recycling’, in which the objects are materially or energetically recycled following a certain period of use. Wood-PHA composites offer a unique solution by providing a WPC where both the reinforcement and the matrix ingredients are entirely biosourced and also biodegradable. 

The physical and mechanical properties of PHA biopolymers are particularly suitable for the manufacture of WPCs. PHAs exhibit a low melt viscosity. PP and PE grades suitable for injection moulding and extrusion typically exhibit a Melt Flow Index (MFI) in the range of 1 to 10 g/10 min at 230 °C, whereas PHB and PHBV grades of similar molecular weight (*M_w_*), have been reported to have an MFI of 14.7 g/10 min at only 190 °C. During extrusion processing with wood fibres, a higher MFI can lead to a better impregnation of the fibres (wetting) and reduce the amount of mixing and shear intensity required for a homogenous fibre distribution. Initial developments on Wood-PHA composites [20,21,22,23] indicate that properties similar to commercially available PP and PE based WPCs are readily achievable. Table 1 summarises the mechanical properties of Wood-PHA composites in early development, compared to commercially available PE- and PP-based WPC products, showing that the tensile strength and tensile moduli were similar. These early results are promising, especially considering the absence of added compatibilisers, coupling agents, stabilizers, or any other functional additives in Wood-PHA composites. It is expected that commercialisation of Wood-PHA composites through the addition of additives, and industrial-scale process optimisation will further improve these mechanical properties [14]. However, current limiting factors in mechanical properties are an exhibited lower strain at failure, toughness and the resulting lower impact strength, as shown in Table 1. Nevertheless, these properties can be improved by incorporating existing solutions, such as the addition of plasticisers, rubber toughening particles, or the use of novel nano-reinforcements, such as cellulose nanofibers (CNF). Such property improvements within specific application contexts are key for expanding the use of Wood-PHA composites, allowing them to gain a significant market share.

## 3. Markets and Production Capacities for Wood-PHA Composites

### 3.1. Applications and Markets for Wood-PHA Composites

**Figure 3:**
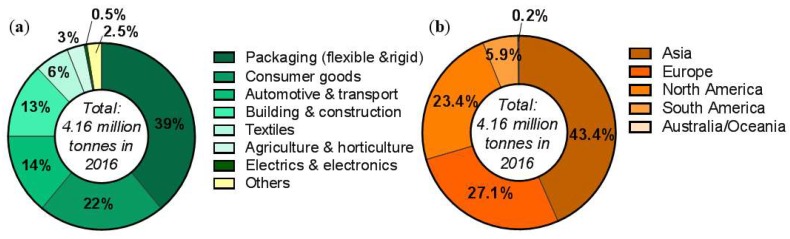
Global production capacities of bioplastics. (**a**) by market segment [25]; and (**b**) by region [3]. Data from European Bioplastics, Nova-Institute (2016).

In 2016, the worldwide production capacity of bioplastics was reported to be 4.16 million tonnes/year [24], distributed across various markets but predominantly in packaging, consumer goods, and automotive sectors, as shown in Figure 3a. The main region for the production of biopolymers is Asia (43%), where production levels are almost as much as Europe and North America combined (at 50%) (Figure 3b). From this 4.16 million tonnes/year market, the production of PHAs represents only 1.6% [3], and this reflects the PHA industrialisation infancy. 

**Figure 4:**
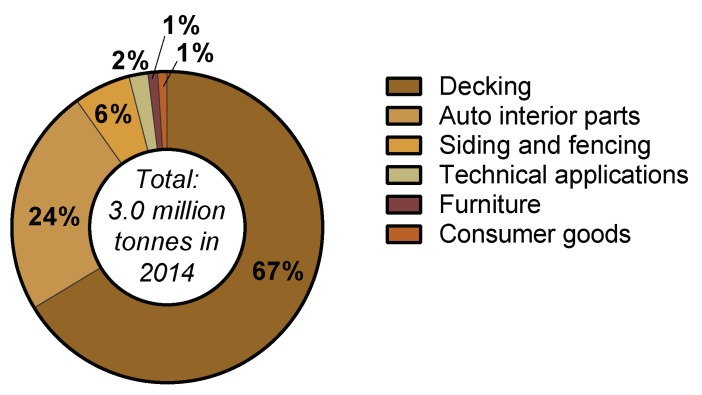
Market segment of Wood Plastic Composites (WPC) in Europe (2012) [26].

The steadily growing WPC market is similar in volume to the global bioplastic market, and stood at 3.0 million tonnes/year in 2014 [4]. In Europe, the WPC market combined with the Natural Fibre Composites (NFC) market represents 15% of the total composite production (which includes glass and carbon synthetic fibres) [26]. This is a significant market share, and the growth of the WPC market al.one, forecasted at a compound annual growth rate (CAGR) of 10.5% [4], is less reliant on government mandated regulations and incentives compared to the bioplastics market in general. Applications for the WPC market differ noticeably compared to the bioplastics market. Decking (67%) and automotive interior parts (24%) are principal applications, as illustrated in Figure 4. However, technical applications, consumer goods, and furniture have been reported to exhibit the highest growth rates [27]. Considering attractive mechanical properties of early Wood-PHA Composites (Table 1), potential applications for these novel materials include automotive interior parts, consumer goods, furniture, and technical applications, which represent 28% of the total WPC market. Long-term performance in a range of environmental conditions is a focus of ongoing research, thus applications involving exposed outdoor environments (decking, siding, fencing, etc.) are not yet suitable to be included in the evaluation of the potential markets. Therefore, the potential market for Wood-PHA composites can be estimated to be around 840,000 tonnes/year (28% of the total WPC market). The market share will be more dependent on the price of PHA compared to PP and PE, if the use is to compete simply as a drop-in substitute. Therefore, in these cases, an important factor is the political framework, such as government legislation or incentives where the downstream benefits of biopolymers are also considered in the equation of cost. For bioplastics, the correlation between political framework and market success is very high. A positive framework can help to encourage initial market growth and investment, whereas a negative setting will put successful developments at stake. In 2011, Italy introduced a ban on plastic bags [28], and today it is the highest consumer of bioplastics in Europe [29]. France adopted the ban on plastic bags in 2016, and has now become the first country to ban plastic cups, plates and cutlery, by 2020 [30]. With these increasing trends to ban selected petroleum-derived products, conventional WPCs are likely to face similar application challenges in the coming decades. We postulate that the actual market share for wood-PHA composites will strongly depend on government legislation in addition to technological advances in PHA production.

The current top three applications for biodegradable polymer in the EU are plastic bags, rigid packaging, and disposable tableware [29]. The last two applications are particularly suitable for Wood-PHA composites, which exhibit a high stiffness. In addition, wood-PHA composites have a unique advantage of being 100% renewable and truly biodegradable WPCs. This offers an attractive solution for bio-based products, particularly for large companies, as it can act to promote sustainability. IKEA (Älmhult, Sweden) is one example of a large multinational company to have highlighted a commitment for their plastic materials to be 100% renewable and/or recycled by 2020 (UN Climate Summit, 2016). As part of this commitment, IKEA have recently signed a £10 billion agreement with a PHA producer (Newlight Technologies, Irvine, CA, USA), with an offtake promise to purchase 50% of the AirCarbon^TM^ material from Newlight’s 23,000 tonnes/year plant in the USA [31]. The use of PHAs in the IKEA product line will start with their home furnishing products, which represent approximately 40% of the total plastic volume used across IKEA [31]. In terms of WPCs, IKEA has already produced injection moulded WPC products, such as chairs [32], which represents a natural opportunity for wood-PHA composites materials. An additional partnership between Newlight Technologies and The Body Shop (London, United Kingdom), a large international cosmetics company, will aim at introducing PHA in The Body Shop’s packaging range [33]. Other key applications, where advanced niche products have been showcased, include a lamp by the Italian designer company FLOS (Merano, Italy), made from Minerv^®^ PHA, developed by Bio-on in Bologna, Italy [34]. Bio-on is further developing and licensing the production of their PHA grade for a wide range of applications, including novel milk carton packaging from renewable origin and fully biodegradable [35]. Toys are also a relevant target for WPCs. In this sector, Lego (Billund, Denmark), the largest toy manufacturer in the world, announced in 2016 an investment of US$150 million, for a bio-based polymer solution to replace their current petroleum-based acrylonitrile butadiene styrene (ABS) plastic [36]. This series of announcements strongly suggests that there is coherent interest towards a goal to use bio-polymers like PHA and move away from petroleum-based plastics. We consider that the involvement of large international companies and supportive government legislation are two key factors that can facilitate market entry of PHAs, increasing the potential for growth in production capacity and consequently allowing for the development of wood-PHA composites.

### 3.2. Production Capacities for PHA and Wood-PHA Composites

**Figure 5:**
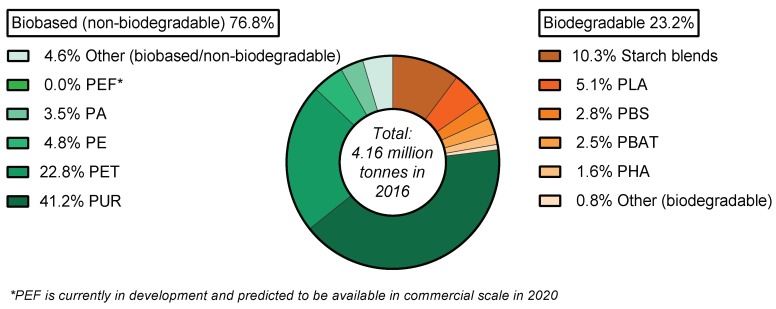
Global production capacity of bioplastics in 2016 [24]. Source: European Bioplastics, Nova-Institute (2016).

The commercial production capacity of PHAs saw a steep increase following the rise in oil prices in 2003. Since then new plants opened in China, US, Italy and Brazil [5]. The current worldwide PHA production capacity (2016) is estimated at 66,000 tonnes/year (representing 1.6% of the global 4.16 million tonnes/year of bioplastics production) [3,24]. As a frame of reference, the global production capacity of bio PE (I’m green^TM^, from Braskem, São Paulo, Brazil) is estimated at 200,000 tonnes/year, and other bioplastics production capacities are shown in Figure 5. Production of wood-PHA composites is currently non-existent. A hypothetical usage of the global PHA production capacity for WPC manufacturing (at an average 50 wt % wood content) would lead to 132,000 tonnes/year of wood-PHA composites. This production volume represents only 4.4% of the global WPC market, which is currently growing at a rate of 10.5% [4]. Therefore, the current WPC market already has an ample volume and growth rate to absorb a significant investment in PHA production given meaningful drivers and applications for the biopolymer as the WPC matrix. 

Due to its fledgling market presence, the total production of PHA biopolymer is estimated in terms of capacity. Precisely assessing the actual production that is genuinely delivered to end-users is difficult to estimate with certainty, since most plants only disclose capacity and not production. The list of companies contributing to the estimated total capacity of 66,000 tonnes/year is summarised in Table 2. Production is conducted today by industrial pure culture fermentation; however, the processes vary in terms of substrate choice, bacterial strain, type of PHA, and method of integration. The scientific literature is rich with intent to establish production methods at reduced costs, including but not limited to the use of wastes and industrial residues as feedstocks, the use of mixed culture bioprocess, and the integration of PHA production with wastewater treatment services [37,38,39,40].

Having secured an agreement with IKEA to purchase 50% of their 23,000 tonnes/year production, Newlight Technologies can become a central player to stimulate the growth of the PHA market. A further report indicates a long-term intention to expand production capacity to 453,000 tonnes/year [31]. In addition, Newlight Technologies have recently signed a 15-year production license agreement with Paques Holdings (Balk, The Netherlands), allowing Paques to manufacture, process and sell PHA at a rate of up to 1.3 million metric tons per year [41]. 

Danimer Scientific (previously Meredian Holdings Group Inc. MHG, Bainbridge, GA, USA), purchased the full set of patents from the P&G company (Cincinnati, OH, USA) to develop a branched PHB polymer, which disrupts crystal formation, allowing for a more flexible and tougher material. Their product Nodax^TM^ is available as foams, fibres or nonwovens, films and latex among others [42]. Their pilot plant in Bainbridge produces 13,600 tonnes/year of PHA, with construction beginning at the site for a plant that will produce 91,000 tonnes/year of PHA [43].

Bio-on (Bologna, Italy) is an intellectual property company, developing PHB polymers from sugar beet and granting licenses worldwide. In 2015, an agreement was signed with Cristal Union, a sugar production company in Aube, France, to open a 5000 tonnes/year production site (expandable to 10,000 tonnes/year) operational by 2018 [44]. More recently (December 2016), an additional agreement worth €55 million with a large multinational client was announced, which will see Bio-on construct a series of plants in Europe and Asia for an overall output of 100,000 tonnes/year [45].

In China, a series of companies, including Tianjin GreenBio Materials (Tianjin, China), Ecomann Biotechnology (Shenzhen, China), and TianAn Biopolymers (Nigbo, China) are reported to be building significant production capacity [46,47,48]. Their combined capacity is at least 15,000 tonnes/year, with materials ranging from P3HB, PHBV (TianAn Biopolymers), to P(3HB-*co*-4HB) copolymers (Tianjin GreenBio), also available in foams.

Other companies, including Tepha Inc. (Lexington, MA, USA), PolyFerm Canada (Kingston, ON, Canada), and Terra Verdae Bioworks (Edmonton, AB, Canada) have focused on commercialisation of PHA for high-value biomedical applications. Their products range from absorbable sutures to heart valves, scaffolds, and controlled drug release [5,49], and consequently represent smaller, but high value, volumes. The use of PHAs for biomedical applications and their cytotoxicity, noncarcinogenicity and biocompatibility has recently been reviewed in [50].

Yield10 Bioscience, previously known as Metabolix (Woburn, MA, USA), made a significant investment in the production of PHA from corn sugars, and in 2009 announced the opening of the largest PHA plant with a production capacity 50,000 tonnes/year. However, in 2016, a decision was made to close their biopolymer activities, and their intellectual property and assets were sold in 2016 to an affiliate of CJ CheilJedang Corporation (Seoul, South Korea) [51]. 

Overall, production of PHA is found to be distributed across the USA, China, and Europe. With regards to actual production, and based on the limited commercial availability of PHA-based products, it appears that most companies are producing below capacity, meaning that overall production levels can be considered to be conservatively overestimated. PHA producers are being cautious about expanding their production capacities, to ensure that investment matches demand and consequently returns. During the initial stages of biopolymer developments, small capacities and volumes are typically associated with high costs, creating stronger incentives for large-scale production when this jump becomes feasible within the market. This is a general issue and is valid for any new material or product that has become large in the market in the past. In the case of PHA, we have previously mentioned that the main two commercialisation barriers are: (1) higher production cost of PHA compared to petroleum-based polymers (indirectly to oil prices), and (2) uncertain market adoption (which can be bolstered by government incentives). Initial risk for these materials can be due to the attempt to compete directly as a drop-in with established products and markets with mature and reliable supply chains. Disruptive technologies can gain market entry and initial growth through providing products and services that, of their nature, offer a unique benefit without being in direct economic competition [12]. 

The costs of producing PHA remain a main barrier to commercialisation within the traditional polymer application space. The price of PHA is dependent on the scale, choice of substrate, bacterial strain, and the type of PHA. For instance, an economic evaluation of PHB production by fermentation with the recovery method of surfactant-hypochlorite digestion was reported to result in a final price of 5.58 US$/kg for an annual production of 2850 tonnes/year. As the production scale increased to 1 million tonnes/year, the price of PHB dropped to 4.75 US$/kg [58]. The cost of substrate was also reported to significantly affect the overall economics of PHA production [59]. In the case of PHBV copolymer, a study, specific to a type of bacteria, revealed that the production cost of P(3HB-*co*-3HV) increased linearly with the increase in HV fraction, from 4.3 to 5.7 US$/kg, for 3HV fractions from 5 to 50 mol % [60]. This is to emphasize the difficulty of comparing PHA prices, due to the complexity of specific PHA polymer types and purities within the PHA family. The costs requirements for a successful market entry of PHA cannot be strictly defined, since these will depend on the market and volumes, juxtaposed the context of the application and the relationships in the value chain. For biomedical applications, where biocompatibility and a high purity are critical, a market price above 10 US$/kg is acceptable. For properties similar to commodity polymers, a cost comparable to PP or PE would be expected, which is of the order of 1.6 US$/kg. Currently, the cost of PHBV, with mechanical properties similar to PP, is about double that of PP and PE, though the emerging producers, including Newlight Technologies, claim PHA can be produced at a price lower than petroleum-based plastics. This would suggest that PHAs have the potential to compete with petroleum-based plastic on price, and consequently gain significant share of their market. Notwithstanding the value of the material in the context of the application will most likely provide for a more stable business model. The cost of the material can also be made by compounding, wherein the fraction of PHA in the composite is reduced but it is still vital to the target of the material properties. Combining PHA with wood reinforcements for the production of WPCs would help in this way to reduce the cost by at least 40%, and provide a unique selling point as a biodegradable WPC. A preliminary economic assessment is essential to evaluate the advantages of this strategic approach. 

## 4. Economic Assessment of Wood-PHA Composites

An economic assessment was conducted to investigate the cost benefits of producing wood-PHA composites, according to two different scenarios: (1) a wood-PHA composite with 50 wt % wood content, based on existing mechanical properties, and (2) a wood-PHA composite based on expected properties with a 60 wt % wood content. Both scenarios are compared to the production of a neat PHA product. Petroleum-based WPCs, which have benefited from several decades of development, are currently commercially available with wood contents typically around 50 wt % and 60 wt % [17], and can be as high as 70 wt % [18]. Various reports and claims on the cost of PHA lead towards a speculative price per kg in the range of approximately 2–8 US$/kg. In 2009, Metabolix was selling PHA pellets at 5 US$/kg [5,10]. Today, PHA pellets are being sold by TianAn Biopolymers at approximately 6.8 US$/kg, and Newlight Technologies claims to be able to produce them in the range of 2–3 US$/kg. For the purpose of this economic assessment, the cost of PHA is assumed to be 7 US$/kg (a conservative, yet realistic value). In comparison, the cost of PP and PE-HD (high density) is reported to be around 1.6 US$/kg, and 1.7 US$/kg respectively [61]. The cost of wood fibre reinforcement is estimated at 0.4 US$/kg. Thus wood-PHA composites with 50 wt % and 60 wt % wood contents, as shown in Table 3, would result in a raw material cost of 3.7 US$/kg and 3.0 US$/kg, respectively. 

Wood fibres being hollow, their apparent density is highly dependent on their state of collapse, and can vary from approximately 0.4 to 1.1 g/cm^3^. In this assessment, radiata pine thermomechanical pulp fibres are considered to be almost fully collapsed after the extrusion process, and exhibit a density of 1.0 g/cm^3^ (density of the fibre wall is 1.15 g/cm^3^). The density of PHA biopolymer is 1.25 g/cm^3^. Consequently, the final densities of a 50 wt % and a 60 wt % wood-PHA composite are equal to 1.11 g/cm^3^ and 1.08 g/cm^3^, respectively (Table 3). Thus, at equivalent weight, a 50 wt % wood content wood-PHA composite is capable of exhibiting a significantly higher stiffness (tensile modulus), as per Table 1, when compared to neat PHA. In product design, specific mechanical properties are often used to evaluate the amount of the material required in order to achieve a given performance. At equal performance, a high-specific-strength material may result in significant cost savings even though the raw material cost per kg is higher, since less material is required. Table 4 lists the specific properties of a 50 wt % wood content wood-PHA composite in comparison to neat PHA. 

As shown in Table 4, a 50 wt % wood content wood-PHA composite exhibits identical specific strength compared to neat PHA material, and a 250% increase in specific stiffness. However, an 85% reduction in strain at failure is observed. The economic assessment for a final product (excluding manufacturing costs) can be approached using two considerations. A first case is one in which wood-PHA material is introduced as a drop-in solution. Existing processing moulds will be used, and the final product will have an identical geometry and volume to the original product, with matching properties. The second case involves the design of a new product to meet specific performance stiffness requirements. This case is only valid for applications where stiffness is the driving factor for the product’s performance, such as rigid packaging, disposable tableware or biodegradable plant pots. Considering both scenarios of product design, a further reduction in final product cost can be achieved, as shown in Table 3. A wood-PHA product of equal volume to a 7 US$ neat PHA product would cost 3.3 US$ and 2.6 US$ for a 50 wt % and 60 wt % wood content, respectively. This product would have a similar tensile strength, higher stiffness and lower strain at failure, compared to a neat PHA counterpart (as per Table 1). However, in applications where stiffness is the sole driving property, a wood-PHA composite product with identical stiffness to a 7 US$ neat PHA would cost as low as 1.5 US$, which represents only 21% of the neat PHA product cost, and is comparable with the cost of PP (1.6 US$/kg) and PE (1.7 US$/kg) [61]. 

Given the overall higher specific properties of wood-PHA composites, the addition of 50 wt % or 60 wt % wood fibres can consequently reduce the final cost of a product beyond its raw material costs. Further cost savings could be achieved for a 70 wt % wood content composite; however, this has not been considered in this assessment. Apart from the gain in certain specific properties, the investment risk of developing wood-PHA composites compared to a neat PHA product is in essence reduced since 50 wt % or more of the product is composed of a ‘filler’. 

## 5. Conclusions

Wood-PHA composites are considered to be a promising opportunity for PHA biopolymers, which could result in a reduced product entry cost equal to 21% of neat PHA cost, and benefit from a rapidly growing WPC market. However, the current drawback of wood-PHA composites is a low strain at failure, which results in poor impact strength and this narrows scope for applications. Although the strain at failure of commercially available WPCs has seen improvements over the last decade, these developments are yet to be made for wood-PHA composites. An added challenge is that even if wood-PHA composites bring a 21% cost reduction compared to similar neat PHA products, they still exhibit a higher cost compared to petroleum-based WPCs. Therefore, the motivation for the use of the biobased composites require benefits that are exclusive to these biodegradable materials in their service or in their after-service management.

The next steps for accelerating the commercial development of wood-PHA composite supply and value chains would involve support from government legislation to enforce environmentally friendly products. Upfront price competition does not necessarily consider downstream burdens and so a more expensive drop-in material cannot expect to succeed without a unique selling attribute of customer benefit, or commonly accepted societal need. Improving strain at failure of wood-PHA composites is identified as a principal composite property in order to broaden the application spectrum for these novel materials. Ultimately, we believe the final stages for a successful implementation of wood-PHA composites would require a genuine commitment to produce wood-PHA pellets on a large scale, coupled with a purchase agreement from a long-term end-user. 

## Figures and Tables

**Table 1:** Summary of mechanical properties achieved for Wood-PHA (polyhydroxyalkanoate) Composites, currently being developed, compared to commercial Wood Plastic Composites (WPC) products based on polypropylene (PP) and polylactic acid (PLA).

	Tensile Strength ^1^ (MPa)	Tensile Modulus ^1^ (GPa)	Strain at Failure ^1^ (%)	Impact Strength ^2^ (kJ/m^2^)
*Pure PHA: Commercially available*				
PHBV ENMAT^TM^ Y1000 (2% HV content)	30	2.8	8.0	-
*WPCs: In development*				
Wood-PHA Composites (PHBV + 50% wood content)	27	6.1	1.0	4.0
*WPCs: Commercially available*				
FKUR—Fibrolon^®^ P7550 (PP + 50% wood content)	22	3.3	3.0	7.9
FKUR—Fibrolon^®^ F8530 (PLA + 50% wood content)	34	3.8	3.8	11.7
Jeluplast—PP H50-500-14 (PP + 50% wood content)	32	4.5	1.9	10.6

^1^ Tested according to ASTM D638 for Wood-PHA Composites and ISO 527-2 for commercial products. ^2^ Tested through standardised Charpy Test according to ISO 179-1/1eU.

**Table 2:** Key players in current PHA production capacity, to date (May 2017).

Company	Country	Capacity (t/year)	Feedstock	Brand Name	PHA Type	Refs
Newlight Technologies	USA	23,000	Biogas and CO_2_	AirCarbon™	n.r.	[5,31]
Danimer Scientific (previously MHG)	USA	13,600	Canola oil	Nodax™	n.r.	[5,43]
Bio-On	Italy	10,000	Sugar beet and cane	Minerv^®^	PHB, PHBV	[47,52]
Tianjin GreenBio Materials	China	10,000	Sugars	SoGreen™	P(3HB-*co*-4HB)	[46]
Ecomann Biotechnology	China	3000	Sugars	AmBio^®^	n.r.	[47]
TianAn Biopolymers	China	2000	Corn Sugar	ENMAT™	P3HB, PHBV	[48]
Kaneka	Japan	1000	Vegetable oil	Aonilex^®^	PHH	[47]
PHB Industrial S. A.	Brazil	500	Sugar cane	Biocycle^®^	P3HB, PHBV	[52,53,54]
Biomer	Germany	500	Corn starch	Biomer^®^	P3HB	[52]
Tepha Inc.	USA	<10	Sugars, 4HB precursors	TephaFLEX^®^	P4HB, P(3HB-*co*-4HB)	[5,52]
PolyFerm Canada	Canada	<10	Vegetable oils, sugars	VersaMer^TM^	PHOHHx, PHNHHp,	[5,52]
Terra Verdae Bioworks	Canada	n.r	Methanol	-	n.r	[55]
Yield10 Bioscience (previously, Metabolix ^1^, Monsanto, Zeneca)	USA	n.r.	Corn sugar	Mirel™	P3HB	[5,10]
Mango Materials	USA	n.r	Methane	-	PHB	[56]
SIRIM	Malaysia	n.r.	Palm Oil	-	n.r.	[57]

^1^ in 2016, Metabolix sold its biopolymer IP and assets to CJ CheilJedang.

**Table 3:** Economic assessment for wood-PHA composites compared to neat PHA products.

	Neat PHA Product	Standard Wood-PHA Composite Product	Low PHA-Content Wood-PHA Composite Product
**Materials**			
Composition of raw material by weight	100 wt % PHA	50 wt % PHA 50 wt % wood fibres	40 wt % PHA 60 wt % fibres
Cost of raw material (US$/kg)	7 US$/kg	3.7 US$/kg (53% of initial cost)	3.0 US$/kg (43% of initial cost)
**Final Product**			
Density of raw material (g/cm^3^)	1.25 g/cm^3^	1.11 g/cm^3^	1.08 g/cm^3^
Composition of final product by volume	100 vol % PHA	44.4 vol % PHA 55.6 vol % wood fibres	34.8 vol % PHA 65.2 vol % wood fibres
Cost of a final product of equal volume (800 cm^3^)	7 US$	3.3 US$ (47% of neat PHA cost)	2.6 US$ (37% of neat PHA cost)
Cost of a final product of equal stiffness	7 US$	1.5 US$ (21% of neat PHA cost)	<1.5 US$

**Table 4:** Specific mechanical properties of neat PHA and Wood-PHA Composites (50 wt % wood).

	Specific Tensile Strength (kN·m/kg)	Specific Tensile Modulus (MN·m/kg)	Specific Strain at Failure (%·m3/kg)
PHA ENMATTM	24	2.2	6.4
Wood-PHA Composites (PHBV + 50% wood content)	24	5.5	0.9
